# Comparison between Itraconazole and Cotrimoxazole in the Treatment of Paracoccidiodomycosis

**DOI:** 10.1371/journal.pntd.0002793

**Published:** 2014-04-17

**Authors:** Ricardo de Souza Cavalcante, Tatiane Fernanda Sylvestre, Adriele Dandara Levorato, Lídia Rachel de Carvalho, Rinaldo Poncio Mendes

**Affiliations:** 1 Tropical Diseases Department – Faculdade de Medicina de Botucatu – Universidade Estadual Paulista (UNESP), Botucatu, São Paulo state, Brazil; 2 Instituto de Biociências de Botucatu – UNESP, Botucatu, São Paulo, Brazil; Baylor College of Medicine, United States of America

## Abstract

**Background:**

There are no published reports on studies comparing itraconazole (ITC), sulfamethoxazole-trimethoprim (cotrimoxazole, CMX), and ITC followed by CMX (ITC/CMX) in the treatment of paracoccidiodomycosis. This study aimed to compare the efficacy, effectiveness, safety and time to clinical and serologic cure in paracoccidioidomycosis patients treated with ITC or CMX, the antifungal agents most widely used.

**Methodology:**

A *quasi-experimental* study was performed in 177 patients with a confirmed or probable diagnosis of paracoccidioidomycosis. Treatment was divided into two stages: 1) initial, which was continued until clinical cure was achieved and the erythrocyte sedimentation rate decreased to normal values; 2) complementary, which was continued until serologic cure was achieved. Medians were compared via the Mann-Whitney test, and frequencies were compared via the chi-squared test. The assessment of variables as a function of time was performed using Kaplan-Meier curves and Cox regression. The significance level was established as p≤0.05.

**Principal Findings:**

No difference was found in the efficacy and effectiveness of the initial treatment of 47 individuals given ITC and 130 individuals given CMX; however, the time to clinical cure was shorter in the former compared with the latter group (105 *vs.* 159 days; p = 0.001), specifically in patients with the chronic form. Efficacy and effectiveness of the three regimens were similar in the complementary treatment; however, the time to serologic cure was shorter when ITC (161 days) or CMX (495 days) was used compared with ITC/CMX (881 days) [p = 0.02]. The independent predictors of a shorter time to serologic cure were treatment with ITC [risk ratio = 6.61 (2.01–21.75)] or with CMX [risk ratio = 5.11 (1.91–13.67)]). The prevalence of side effects was lower with ITC (6.4%) than with CMX (20.0%; p = 0.03).

**Conclusions:**

Since ITC induced earlier clinical cure and was better tolerated than CMX, such triazole should be considered the first-choice for PCM treatment.

## Introduction

Paracoccidiodomycosis (PCM) is an endemic mycosis in Latin America caused by dimorphic fungi belonging to the *Paracoccidioides brasiliensis* complex and the *Paracoccidioides lutzii* complex, whose natural habitat is soil [1]. PCM exhibits three main clinical forms: a) acute/subacute, which prevails among children, adolescents, and young adults and affects organs rich in mononuclear phagocyte system elements; b) chronic, which affects adults, mostly rural workers and men, and is characterized by the involvement of the lungs and the upper digestive and airway mucous membranes; and c) residual, which comprises the sequelae remaining after efficacious treatment, especially in the lungs, larynx, and adrenal glands [2].

The appropriate treatment of PCM began 32 years after its initial description by Adolfo Lutz (1908) [3] with sulfapyridine (1940) [4], and was followed by amphotericin B (1958) [5], trimethoprim-sulfamethoxazole combination, called cotrimoxazole – CMX (1973) [6], ketoconazole (1979) [7], [8], itraconazole – ITC (1987) [9], [10], [11], [12] and voriconazole (2007) [13].

Despite the development of these therapeutic agents in the last decades, only four comparative studies have been conducted to date [13], [14], [15], [16]. In 2006, the first Brazilian guidelines on the diagnosis and treatment of PCM was published, recommending ITC and CMX as first- and second-choice agents, respectively [17]. Only two previous studies comparing ITC and CMX in the treatment of PCM patients were performed [18], [19].

The aim of the present study was to compare the efficacy, effectiveness, and safety of ITC and CMX during the initial and complementary treatment of PCM and to compare the time to achieve clinical cure, the return of the erythrocyte sedimentation rate (ESR) and acute-phase reactants to the normal levels, and persistent negativation of the double immunodiffusion (DID) reaction in agar gel.

**Author summary**

Paracoccidiodomycosis (PCM) is an endemic disease in Latin America caused by dimorphic fungi belonging to the *Paracoccidioides* genus that affects mainly rural workers in their most productive period of their lives. There are no published reports on studies comparing itraconazole (ITC), sulfamethoxazole-trimethoprim (cotrimoxazole, CMX), and ITC followed by CMX (ITC/CMX) in the treatment of PCM. The present study compared individuals with PCM treated with ITC or CMX, which are the antifungal agents most widely used in the treatment of this disease. In the initial stage of treatment, the time to clinical cure was shorter in the 47 individuals given ITC compared with the 130 individuals given CMX specifically in the cases of patients with the chronic form of disease. In the complementary treatment stage, the time to serologic cure was shorter when ITC or CMX was used compared with ITC/CMX. The prevalence of side effects was lower when ITC was used compared with CMX. The present study found that ITC induced earlier clinical cure and was better tolerated compared with CMX; thus, ITC could be considered the first-choice antifungal agent for PCM treatment.

## Materials and Methods

A total of 177 individuals with PCM who were seen at the Discipline of Infectious and Parasitic Diseases, Faculdade de Medicina de Botucatu – São Paulo State University (Universidade Estadual Paulista – UNESP) between 1988 and 2012 were analyzed.

The first consultation was performed on a ward, at the triage center, or in the emergency department of the Clinical Hospital by a specialist in infectology in our service, who selected the antifungal regimen. The tests performed upon admission before the beginning of treatment and the periodicity of follow-up visits and laboratory tests were standardized; thus, they were the same for all patients independent of the therapeutic regimen selected. Inpatients were referred to the paracoccidiodomycosis outpatient clinic following hospital discharge, as were also the individuals who were first assisted at the triage center or the emergency department. The consultations performed at the outpatient clinic were systematically supervised by the same physician, who applied a standard protocol.

### Inclusion criteria

The following individuals with PCM were included in the present study: a) confirmed cases, i.e., those with compatible clinical manifestations in whom typical *P. brasiliensis* yeast forms were identified in clinical materials; b) probable cases, i.e., those with compatible clinical manifestations and positive specific serum antibodies on DID in agar gel, but without identification of *P. brasiliensis* [17]; c) treatment-naïve individuals or those who had begun treatment against PCM less than 30 days earlier; d) individuals exhibiting a new episode of disease activity who had a mycologically and/or serologically positive diagnosis and had not used antifungal drugs for at least six months; and e) individuals who were initially treated with ITC or CMX.

### Exclusion criteria

Patients with infectious, parasitic, neoplastic, or inflammatory (connective tissue disorders) systemic diseases as comorbidities; those using antifungal agents for any other reason; those using drugs that interfere with the kinetics of ITC or CMX; and pregnant or breastfeeding women were excluded.

### Classification of clinical forms

The patients were classified according to the clinical form and severity of the disease following Mendes [2], as described below. The acute or subacute form, also known as the juvenile form, is characterized by a short period of history, usually from weeks to a few months. It affects mainly children, adolescents, and young adults, and tends to predominate in organs rich in mononuclear phagocyte system elements, such as lymph nodes, liver, spleen, and bone marrow. The severity of disease was classified in moderate and severe based on the following criteria: a) weight loss greater than 10% of the usual body weight; b) DID titers equal to or higher than 1/64; c) tumor-like or suppurative appearance of the superficial or deep lymph nodes; d) the involvement of organs and systems such as the bones, bowels, adrenal glands, and the central nervous system; and e) negative intradermal reaction to paracoccidioidin. The presence of at least three of those criteria defined a case as severe, while the cases that met up to two criteria were considered moderate. No mild cases were considered in this clinical form. The chronic or adult form is characterized by insidious establishment, usually over four and often six months, patients older than 30 years, and a tendency to affect the lungs and the upper digestive and airway mucous membranes. This form was classified as mild, moderate, or severe, as follows: Severe cases met three or more of the following criteria: a) weight loss greater than 10% of the usual body weight; b) severe lung involvement; c) involvement of other organs and systems, such as the adrenal glands, central nervous system, and bones; d) tumor-like or suppurative involvement of the superficial lymph-nodes; e) DID titers equal to or higher than 1/64; and e) negative intradermal reaction to paracoccidioidin. The moderate cases were those that exhibited weight loss of 5 to 10% of the usual body weight, DID titers of 1/16 or 1/32, and one or two of the criteria described above. The mild cases exhibited less than 5% of weight loss, exclusive involvement of the lungs, upper digestive and airway mucous membranes, or the skin, DID titers below 1/16, and positive intradermal reaction to paracoccidioidine

### Interventions

The interventions to which the patients were subjected were treatment with CMX or ITC. CMX was administered to adults at a dose of 1,440 mg every 12 hours. It was usually administered orally throughout treatment, but in some cases, it was administered first intravenously (IV) and then orally. CMX was given to children in a dose of 4 to 8 mg/kg of trimethoprim orally every 12 hours. In both adults and children, the serum levels of sulfamethoxazole were monitored and kept above 70 µg/kg in the initial stage and above 50 µg/kg in the complementary stage of treatment, with eventual adjustments of the dose. ITC was always administered orally in capsules after a meal at a dose of 200 mg/day once per day for adults and of 5 to 10 mg/kg/day for children. Some patients underwent initial treatment with ITC and complementary treatment with CMX or other sulfamide derivatives, such as sulfadiazine or sulfadoxine. Sulfadiazine was administered orally at a dose of 100 mg/kg/day divided in four equal doses every six hours for a total of 4.0 g/day. Sulfadoxine was administered orally once per week at a dose of 1.0 to 2.0 g.

### Sample size

The sample size was calculated with an α error of 5%, a test power of 80%, a CMX efficacy of 70%, and an ITC efficacy of 95%, based on previously published reports [9], [10], [11], [13], [16], [20] and using the following equation:
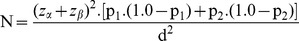
where

N – smallest number of participants in each group;

d – difference between the efficacy of treatments;

p_1_ – proportion of expected therapeutic successes with CMX;

p_2_ – proportion of expected therapeutic successes with ITC.

Thus, the sample size corresponded to at least 33 participants per group. Because criteria in addition to efficacy were also assessed, the sample size was increased.

### Patients' follow-up

Treatment comprised two stages, initial and complementary [21]. Initial treatment was continued until clinical cure was achieved. Clinical cure was defined as the disappearance of the signs and symptoms exhibited by the patients at recruitment, other than those possibly associated with sequelae, and a decrease of ESR to its normal values. In the case of the individuals who did not exhibit increased ESR at recruitment, the variable used to define the end of initial treatment and the beginning of complementary treatment was the return of the serum γ-globulin levels to the normal, as measured via serum protein electrophoresis. During the initial stage of treatment, the patients underwent clinical, radiologic, parasitological, urinary, and serologic assessment and blood biochemistry tests every month.

During the complementary treatment, the participants underwent clinical, serologic, and radiologic assessment every three months for one year after achieving persistently negative serologic tests.

### Laboratory tests

The patients' follow-up included a large number of tests, and the following ones were analyzed in the present study. DID in agar gel, following Restrepo (1972), was the routine serologic test performed [22], and ESR was the standard hematologic parameter assessed. Blood biochemistry included the measurement of the serum levels of total and conjugated bilirubin, aminotransferases, alkaline phosphatase, γ-glutamyl transferase (γ-GT), urea, creatinine, C-reactive protein (CRP), and mucoproteins, later replaced by α_1_–acid glycoprotein, and serum protein electrophoresis. The blood tests were performed at the Laboratory of Hematology of Botucatu, the serologic tests were performed at the Tropical Disease Research Laboratory, Medical Mycology, and the blood biochemistry tests were performed at the Central Laboratory of the Clinics Hospital, Faculdade de Medicina de Botucatu, UNESP. The results of all the tests assessing the hepatobiliary and kidney function are expressed as the ratio of the serum peak found to the upper limit of normality (N), whereby values between N and 2N were considered mild, values between 2N and 3N were considered moderate, and values above 3N were considered intense [23].

In addition to the routine tests included in the follow-up protocol, other assessments were performed as a function of particular clinical indications.

### Study design

The patients were allocated to four groups according to the antifungal drugs used during the initial and complementary treatment, as follows: G_1_ – Participants who used ITC in the initial and complementary treatments; G_2_ – Participants who used ITC in the initial treatment and CMX in the complementary treatment; G_3_ – Participants who used ITC in the initial treatment and sulfamide derivatives in the complementary treatment; G_4_ – Participants who used CMX in the initial and complementary treatments.

Each patient's individual treatment was selected by the specialist in infectology who performed the first assessment, with no influence whatsoever from either the patients or the investigators. Because the participants were not randomized, this was a *quasi*-experimental study.

As the number of participants in Group G_3_ was small, it was included in the analysis of the initial treatment only.

**Assessment of homogeneity**. The homogeneity of the groups that used ITC (G_1_, G_2_, and G_3_ together) or CMX (G_4_) as the initial treatment was assessed based on the patients' age, gender, clinical form, and severity of disease.

### Outcomes

The following definitions were used in the analysis of outcomes: (1) Clinical cure – disappearance of the signs and symptoms present before the onset of antifungal treatment, except for residual manifestations related to sequelae; (2) Serologic cure – persistently negative results for specific antibodies assessed via DID in agar gel for more than one year; the date of the first negative serologic result was considered the reference for estimating time to seroconversion; (3) Therapeutic failure – the persistence or recrudescence of symptomatology after onset of antifungal treatment or the persistence of any titer of serum specific antibodies measured by DID; (4) Apparent cure – clinical and serologic cure for more than two years after treatment discontinuance; (5) Side effects – adverse events reported by the patients or found in laboratory exams; (6) Therapeutic success – clinical and serologic cure and the return of ESR or γ-globulin to their normal values; 7) Efficacy – percent frequency of therapeutic successes, assessed only in the participants who received adequate treatment; and (8) Effectiveness – percent frequency of therapeutic successes taking all the study participants into account, thus corresponding to an intention-to-treat analysis.

The following outcomes were established:

1. Primary outcomes. First – assessment of initial treatment: efficacy, effectiveness, time to clinical cure and time to return to normal ESR values; Second – assessment of complementary treatment: efficacy, effectiveness and time to serologic cure; Third – assessment of apparent cure: frequency of patients who met the criteria for apparent cure; Fourth – assessment of treatment safety: frequency of side effects detected by means of clinical manifestations or alterations in laboratory tests.
2. Secondary outcomes. First – pairwise comparison of the sensitivity of ERS and markers of active inflammation (CRP, α_1_-acid glycoprotein, and γ-globulin) according to the clinical form of disease; Second – comparison of the time to return to normal values of acute-phase reactants values according to the clinical form of disease and antifungal agent used.

### Ethics statement

The present study was assessed and approved by the research ethics committee of Faculdade de Medicina de Botucatu – São Paulo State University. Written informed consent for participation was given by the patient or parents.

### Statistical analysis of results

The continuous variables are expressed as median, 1^st^, and 3^rd^ quartiles. Those variables were compared using the Mann-Whitney test for independent variables. The Kaplan-Meier curve was used in the analysis of variables as a function of time, and Cox regression was used in the assessment of risk factors. The categorical variables are expressed as percentages and were compared using the χ^2^ test or Fisher's exact test. Those analyses were performed following Zar's specifications [24]. The statistical analyses were performed using software SAS Version 9.3. The null hypothesis was rejected with an error equal to or lower than 5% in a two-tailed test. Values between 5% and 10% were interpreted as tendency.

## Results

**Figure 1:**
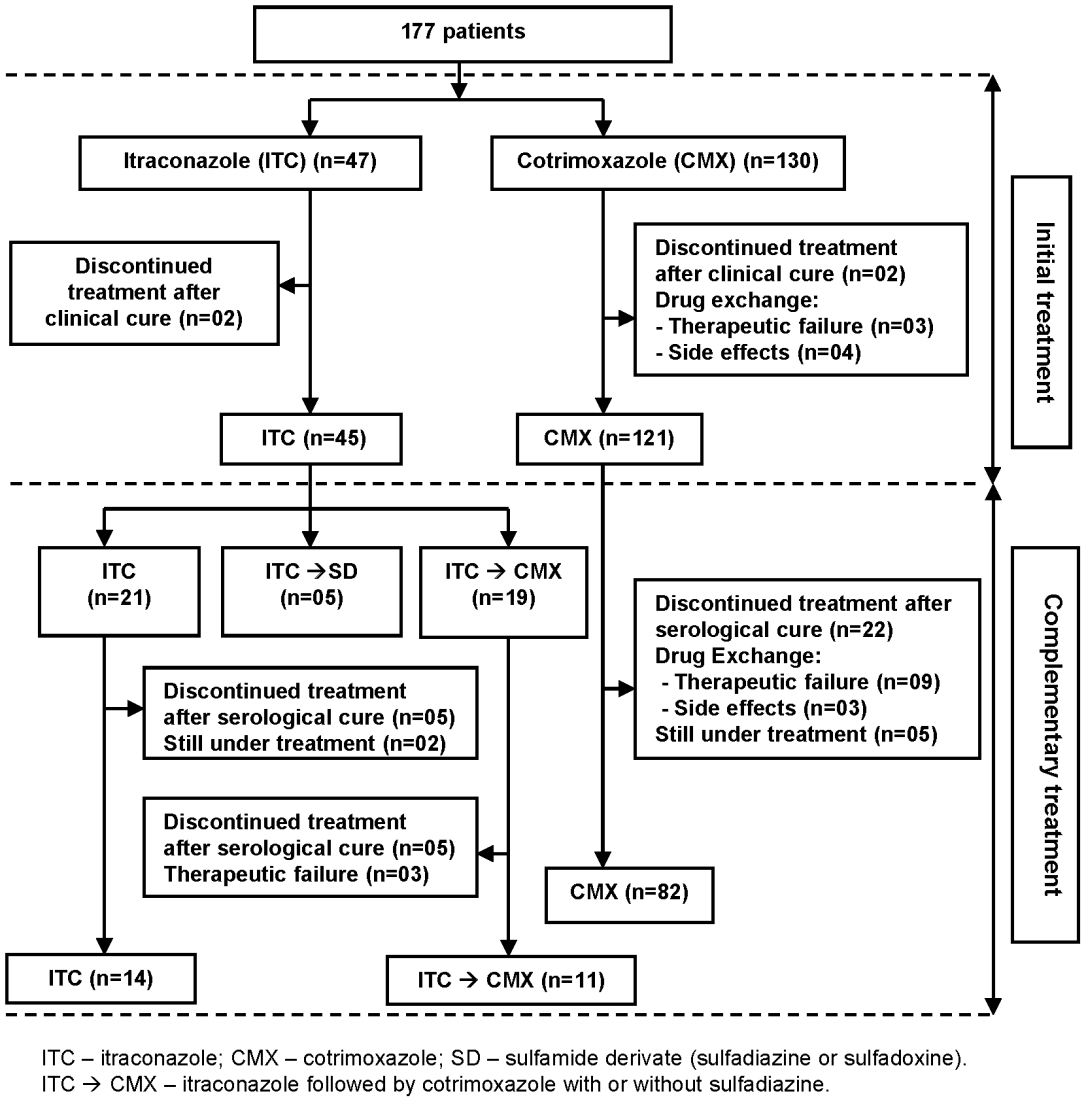
Flowchart of the allocation of 177 individuals with paracoccidiodomycosis according to treatment and time-point of outcome analysis.

A total of 177 individuals met the study inclusion criteria. Figure 1 depicts the flowchart of allocation of patients according to therapeutic regimen and the time-points of outcome assessment.

### Primary outcomes

**1. First primary outcome – Initial treatment.** The patients who used ITC as an initial treatment did not differ from those who used CMX as the initial treatment with regard to age, gender, or clinical form of disease (table 1).

**Table 1:** Distribution of 177 individuals with paracoccidiodomycosis subjected to initial and complementary treatment according to epidemiological, clinical, and serologic features.

		Initial treatment		p value	Complementary treatment			p value
		ITC (n = 47)	CMX (n = 130)		ITC (n = 21)	ITC/CMX (n = 19)	CMX (n = 121)	
Age (years)		43,0 [33,5–53,8]	45,5 [35,0–54,0]	0,65	53,0 [42,0–60,0]^a^	37,0 [32,0–42,0]^c^	45,5 [34,0–54,0]^b^	0,002
Male gender (%)		87,2	88,5	0,82	84,2	89,5	87,9	0,93
Clinical form (%)				0,63				0,25
	Acute	29,8	26,2		21,1	42,1	27,6	
	Chronic	70,2	73,8		78,9	57,9	72,4	
Acute form (%)				0,65				0,45
	Moderate	0,0	3,1		0,0	0,0	3,4	
	Severe	29,8	23,1		21,1	42,1	24,2	
Chronic form (%)				0,65				0,57
	Mild	6,4	4,6		5,3	10,3	5,2	
	Moderate	51,1	56,2		57,9	31,6	54,8	
	Severe	12,8	13,1		15,8	15,8	12,9	
Initial DID (1:)		16 [2–64]	16 [4–64]	0,38	4 [2–16]^c^	64 [8–128]^a^	16 [4–64]^ab^	0,03

ITC – itraconazole; CMX – cotrimoxazole; SD – sulfamide derivate (sulfadiazine or sulfadoxine). ITC→CMX – itraconazole followed by cotrimoxazole with or without sulfadiazine. Comparison of medians: Mann-Whitney test; data expressed as median, 1^st^, and 3^rd^ quartiles. Comparison of frequencies: chi-square test.

**Figure 2:**
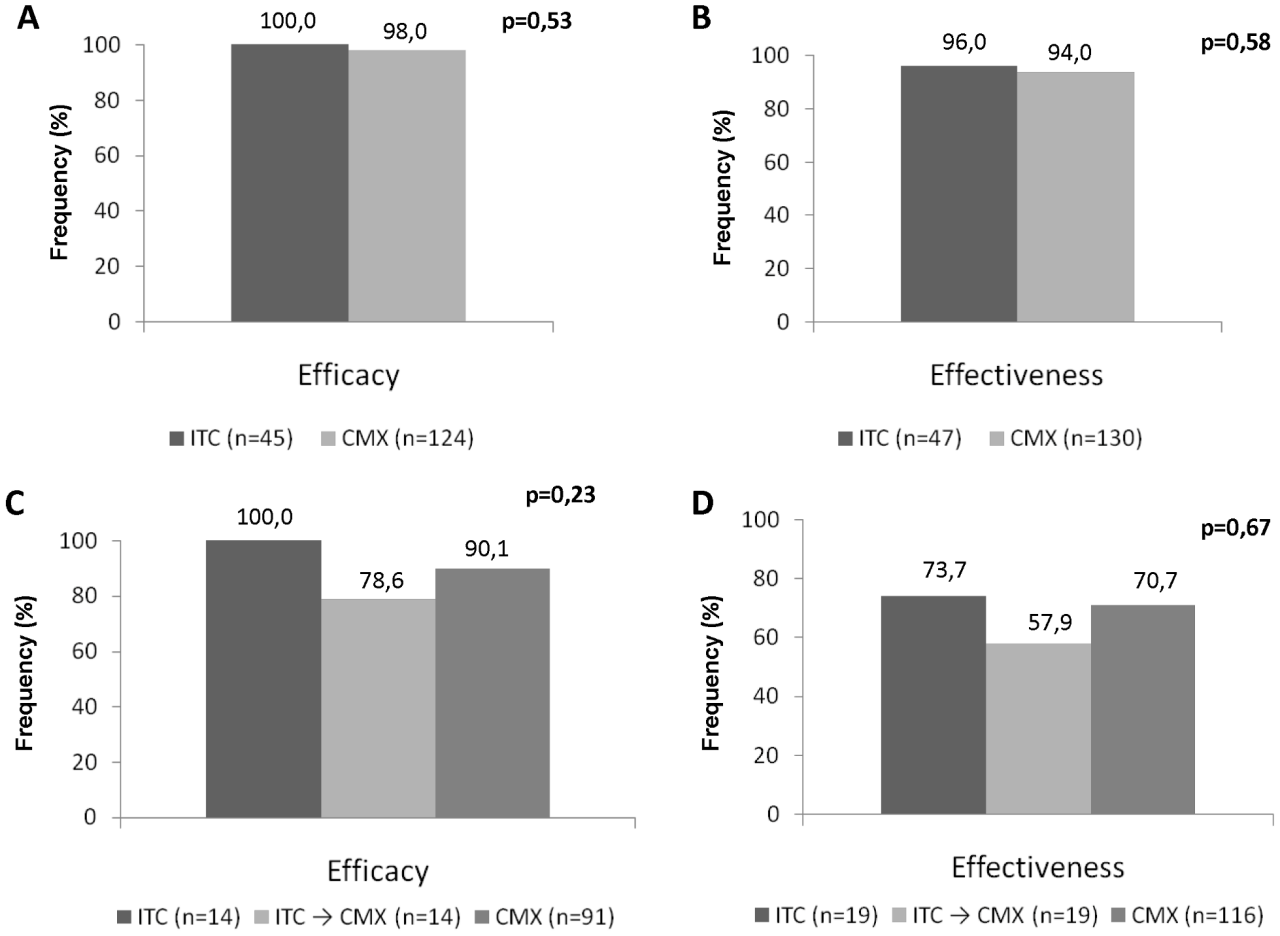
Efficacy (A) and effectiveness (B) of initial treatment in 177 individuals with paracoccidiodomycosis according to the therapeutic regimen used, and efficacy (C) and effectiveness (D) of complementary treatment in 138 individuals with paracoccidiodomycosis according to the therapeutic regimen used.

**Figure 3:**
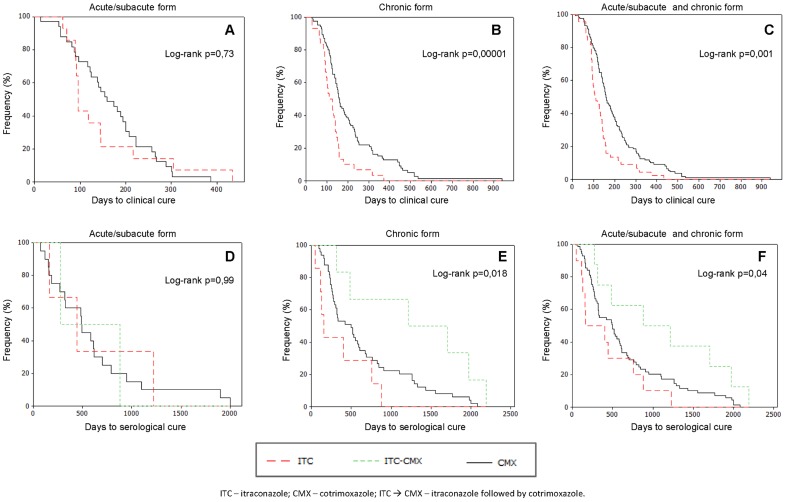
Kaplan-Meier curves assessing the time to clinical and serologic cure in individuals with paracoccidiodomycosis according to the antifungal agent used and the clinical form of disease. A–C: assessment of clinical cure in 177 individuals - (A) acute/subacute form; (B) chronic form; (C) acute/subacute and chronic forms. D–F: assessment of serologic cure in 138 individuals - (D) acute/subacute form; (E) chronic form; (F) acute/subacute and chronic forms.

ITC and CMX exhibited the same efficacy and effectiveness for the initial treatment of PCM [p>0.05] (figure 2). The patients given ITC as the initial treatment achieved clinical cure in a shorter time (105 days) compared with those treated with CMX (159 days) [p = 0.001]. The time to clinical cure did not differ as a function of the therapeutic agent (p>0.05) in the individuals with the acute/subacute form of disease, but among the patients with the chronic form, the time to clinical cure was shorter in those treated with ITC compared to CMX [112 *vs.* 159 days; p<0.001] (table 2, figure 3). The time to return to normal ESR values did not differ as a function of the treatment performed [p>0.05] (table 3).

**Table 2:** Kaplan-Meier analysis comparing the time in days to clinical and serologic cure in 177 and 138 individuals with paracoccidiodomycosis, respectively, according to the antifungal agent used and the clinical form of disease.

Primary variable	Secondary variable	Tertiary variable	Crossings	p value	Interpretation
Time to clinical cure	Treatment	Acute	ITC (96) *vs* CMX (159)	0,73	ITC = CMX
		Chronic	ITC (112) *vs* CMX (159)	0,00001	ITC<CMX
		Acute and chronic	ITC (105) *vs* CMX (159)	0,001	ITC<CMX
	Clinical form		MA *vs* SA *vs* MC *vs* MdC *vs* SC	0,001	
			MA (71) *vs* SA (159)	0,001	MA<SA
			MA (71) *vs* MC (76)	0,48	MA = MC
			MA (71) *vs* MdC (152)	0,00001	MA<MdC
			MA (71) *vs* SC (196)	0,00001	MA<SC
			SA (159) *vs* MC (76)	0,00001	SA>MC
			SA (159) *vs* MdC (152) *vs* SC (196)	0,12	SA = MdC = SC
			MC (76) *vs* SC (196)	0,00001	MC<SC
			MC (76) *vs* MdC (152)	0,00001	MC<MdC
Time to serological cure	Treatment	Acute	ITC (441) *vs* CMX (488) *vs* ITC/CMX (273)	0,99	ITC = CMX = ITC/CMX
		Chronic	ITC (159) *vs* CMX (497) *vs* ITC/CMX (1217)	0,018	(ITC = CMX)<ITC/CMX
		Acute and chronic	ITC (161) *vs* CMX (495) *vs* ITC/CMX (881)	<0,05	(ITC = CMX)<ITC/CMX
	Clinical form		MA *vs* SA *vs* MC *vs* MdC *vs* SC	0,04	
			MA (157) *vs* SA (495)	0,027	MA<SA
			MA (157) *vs* MC (159)	0,65	MA = MC
			MA (157) *vs* MdC (425)	0,023	MA<MdC
			MA (157) *vs* SC (551)	0,049	MA<SC
			SA (495) vs MC (159)	0,12	SA = MC
			SA (495) *vs* MdC (425) *vs* SC (551)	0,99	SA = MdC = SC
			MC (159) vs SC (551)	0,08	MC = SC
			MC (159) *vs* MdC (425)	0,049	MC<MdC

The time medians are indicated in parentheses. ITC – itraconazole; CMX – cotrimoxazole; ITC/CMX – itraconazole followed by cotrimoxazole; MA – moderate acute form; SA – severe acute form; MC – mild chronic form; MdC – moderate chronic form; SC – severe chronic form.

**Table 3:** Kaplan-Meier analysis comparing the time in days to return to the normal values of erythrocyte sedimentation rate (ESR) and markers of active inflammation in 177 individuals with paracoccidiodomycosis according to the antifungal agent used and the clinical form of disease.

Primary variable	Secondary variable	Tertiary variable	Crossings	p value	Interpretation
Time to normal ERS	Treatment	Acute	ITC (145) *vs* CMX (117)	0,52	ITC = CMX
		Chronic	ITC (95) *vs* CMX (116)	0,33	ITC = CMX
		Acute and chronic	ITC (95) *vs* CMX (117)	0,70	IT = CMX
	Clinical form		MA *vs* SA *vs* MC *vs* MdC *vs* SC	0,008	
			MA (39) *vs* SA (138)	0,005	MA<SA
			MA (39) *vs* MC (63)	0,42	MA = MC
			MA (39) *vs* MdC (103)	0,002	MA<MdC
			MA (39) *vs* SC (110)	0,001	MA<SC
			SA (138) *vs* MC (63)	0,13	S = MC
			SA (138) *vs* MdC (103) *vs* SC (110)	0,83	SA = MdC = SC
			MC (63) vs SC (110)	0,19	MC = SC
			MC (63) *vs* MdC (103)	0,19	MC = MdC
Time to normal CRP	Treatment		ITC (97) *vs* CMX (95)	0,93	ITC = CMX
Time to normal Muco	Treatment		ITC (58) *vs* CMX (92)	0,93	ITC = CMX
	Clinical form		MA (99) *vs* SA (92) *vs* MC (21) *vs* MdC (53) *vs* SC (106)	0,38	MA = SA = MC = MdC = SC
Time to normal α1-glycop	Treatment		ITC (70) *vs* CMX (103)	0,55	ITC = CMX
Time to normal γ-glob	Treatment		ITC (158) *vs* CMX (175)	0,96	ITC = CMX
	Clinical forms		MA (99) *vs* SA (187) *vs* MC (63) *vs* MdC (161) *vs* SC (142)	0,07	MA = SA = MC = MdC = SC

The time medians are indicated in parentheses. ESR – erythrocyte sedimentation rate; CRP – C-reactive protein; Muco – mucoprotein; α1-gliycop – α1-acid glycoprotein; γ-glob – γ-globulin; ITC – itraconazole; CMX – cotrimoxazole; ITC/CMX – itraconazole followed by cotrimoxazole; MA – moderate acute form; SA – severe acute form; MC – mild chronic form; MdC – moderate chronic form; SC – severe chronic form.

**2. Second primary outcome – Complementary treatment**. The patients treated with ITC only were older than those who used CMX, and those who used CMX were older than those given ITC followed by CMX. The groups did not differ in terms of gender, clinical form, or disease severity (table 1). The levels of serum antibodies were higher in the individuals who used ITC followed by CMX than in those treated with ITC alone. The serum antibody levels did not differ between the patients who used CMX and those who used ITC followed by CMX, and they were higher in the individuals who used CMX alone compared with those treated with ITC alone (table 1).

No difference was found in the efficacy and effectiveness of complementary treatment with ITC alone, CMX alone, or ITC followed by CMX (Figure 2).

The time to achieve serologic cure did not differ between the groups treated with ITC alone (median = 161 days) and CMX alone (median = 495 days) [p = 0.13], but it was shorter in both cases compared with the group given ITC in the initial treatment followed by CMX in the complementary treatment (median = 881 days) [p = 0.02]. That same result was found in the case of the patients with chronic PCM, but not those with the acute form of disease (table 2; figure 3). Multivariate analysis using Cox regression showed that treatment with ITC or CMX alone was predictive of a shorter time to serologic cure compared with the regimen using ITC followed by CMX (table 4).

**Table 4:** Multivariate analysis performed to identify predictors of the time to serologic cure in 138 individuals with paracoccidoidomycosis.

		Hazard ratio (95%CI)	p value
Age		1,003 (0,985–1,023)	0,72
Acute *vs* chronic forms		0,370 (0,123–1,110)	0,07
Initial DID		0,910 (0,822–1,008)	0,07
Time to clinical cure		0,998 (0,996–1,000)	0,06
Clinical forms*			
	Severe acute	0,422 (0,134–1,331)	0,14
	Mild chronic	4,152 (1,452–11,873)	0,008
	Severe chronic	1,618 (0,726–3,602)	0,23
Treatment**			
	Itraconazole	6,615 (2,012–21,749)	0,009
	Cotrimoxazole	5,110 (1,910–13,668)	0,001

DID – immunodiffusion in agar gel specific for *P. brasiliensis*.

CI - confidence interval.

* Relative to the clinical forms, the moderate chronic form was considered the reference in the assessment of the time to serologic cure.

** Relative to the treatment regimens, itraconazole followed by cotrimoxazole was considered the reference in the assessment of the time to serologic cure.

**3. Third primary outcome – Apparent cure**. The prevalence of patients who achieved apparent cure was high: 92.9% of those treated with ITC alone, 95.1% of those treated with CMX alone, and 100.0% of those treated with ITC followed by CMX, without significant difference among the groups (p>0.05).

**4. Fourth primary outcome – Assessment of treatment safety**. The incidence of clinical complaints after the onset of initial treatment was lower in the individuals treated with ITC (6.4%) compared with those treated with CMX (20.0%) [p = 0.03]. Epigastric pain was more frequent in the patients given CMX (13.8%) compared with those given ITC (2.1%) [p = 0.05]. Headache (2.1%) and dizziness (2.1%) were reported by the individuals who used ITC, while diarrhea (3.8%), vomiting (0.8%), and skin rashes (0.8%) occurred in those who used CMX. Both antifungal agents were associated with hepatobiliary and kidney function disorders; direct bilirubin was the only parameter that exhibited significant difference, being more frequently altered in the patients who took ITC (34.6%) compared with those who took CMX (3.6%) [p<0.001] (table 5). As a rule, the intensity of the alterations was mild, except in the case of γ–GT in the individuals treated with ITC, who exhibited a median increase of 6.5 times the upper limit of normality. Comparisons among the groups showed only a tendency toward greater elevation of γ–GT in the patients treated with ITC compared with those treated with CMX (p = 0.07).

**Table 5:** Blood chemistry alterations secondary to the use of antifungal agents in 177 individuals with paracoccidiodomycosis.

	Disturbance (%)		p value	Magnitude of disturbance		p value
	ITC	CMX		ITC	CMX	
AST	11,5	17,6	0,34	1,2 [1,1–6,8]	1,3 [1,1–1,5]	0,85
ALT	19,2	29,4	0,44	1,5 [1,5–3,1]	1,5 [1,2–2,2]	0,59
ALP	11,5	16,9	0,38	1,7 [1,1–2,1]	1,2 [1,2–1,5]	0,80
γ – GT	11,5	11,8	0,63	6,5 [1,5–6,6]	1,3 [1,1–1,7]	0,07
TB	7,7	2,4	0,23	1,3 [1,2–1,4]	2,7 [1,9–3,5]	0,12
DB	34,6	3,6	<0,001	2,0 [1,7–4,0]	3,3 [2,3–16,7]	0,30
Urea	10,3	15,1	0,38	1,2 [1,1–1,5]	1,1 [1,1–1,2]	0,41
Creatinine	6,9	9,7	0,48	1,1 [1,1–1,1]	1,1 [1,1–1,2]	0,63

AST – aspartate aminotransferase; ALT – alanine aminotransferase; γ – GT – gamma-glutamyl transferase; ALP – alkaline phosphatase; TB – total bilirubin; DB – direct bilirubin. Categorical variables were compared using the chi-square test and Fisher's exact test, and the continuous variables were compared using the Mann-Whitney test.

### Secondary outcomes

The sensitivity of ESR and markers of active inflammation is described in tables 6 and 7.

**Table 6:** Sensitivity of the erythrocyte sedimentation rate and markers of active inflammation before the onset of antifungal treatment in 177 individuals with paracoccidiodomycosis.

	Acute/subacute	Chronic	p value	Acute and chronic
ERS	84,8	54,5	<0,01	62,7
C reactive protein	56,5	53,6	0,99	54,4
Mucoprotein	66,7	57,1	0,63	60,0
α1-acid glycoprotein	38,9	47,4	0,72	45,2
γ-globulin	86,7	55,4	<0,01	63,9

ESR – erythrocyte sedimentation rate. Comparison of frequencies: chi-square test.

**Table 7:** Pairwise comparison of the sensitivity of the erythrocyte sedimentation rate and markers of active inflammation and assessment of the concordance degree in 169 individuals with paracoccidiodomycosis.

1° *vs* 2°	Patients (n°)	1° (+) e 2° (+)	1° (−) e 2° (−)	1° (+) e 2° (−)	1° (−) e 2° (+)	p value	Kappa value	95%CI	Degree of confidence
ERS *vs* CRP	79	28	20	16	15	0,99	0,21	0,00–0,42	Mild
ERS *vs* Muco	70	36	14	14	06	0,10	0,37	0,14–0,61	Mild
ERS *vs* γ-glob	166	75	33	27	31	0,70	0,27	0,11–0,42	Mild
CRP *vs* α1-glycop	57	19	24	10	04	0,20	0,51	0,28–0,73	Moderate
CRP *vs* γ-glob	79	25	14	18	22	0,60	0,00	0,00–0,22	Mild
Muco *vs* γ-glob	70	32	13	10	15	0,40	0,27	0,03–0,51	Mild
α1-glycop *vs* γ-glob	73	20	17	13	23	0,10	0,03	0,00–0,25	Mild

ESR – erythrocyte sedimentation rate; CRP – C-reactive protein; Muco – mucoprotein; α1-gliycop – α1-acid glycoprotein; γ-glob – γ-globulin. McNemar test.

Comparisons of the time to return to normal values of ESR and markers of active inflammation did not indicate differences as a function of the therapeutic regimen used, as table 3 shows.

## Discussion

Despite the use of efficacious antifungal treatment, the individuals with PCM have persistent latent *P. brasiliensis* foci, which makes the complementary of treatment necessary until the cell-mediated immunity recovers to prevent disease relapse. As a consequence, antifungal treatment for PCM continues for a long time and is guided by the reduction of serum-specific antibody levels as measured by DID until negativity is achieved. The initial stage of treatment lasts as long as the patient exhibits symptoms and increased ESR. As a rule, clinical cure is achieved before the serologic cure, and this marks the shift to complementary treatment, during which the patients are asymptomatic, but antifungal medication is continued [21]. Because these are very different stages of treatment, they are discussed separately.

The homogeneity exhibited by the groups subjected to initial treatment with ITC or CMX makes the results of the present study consistent. During this stage, the efficacy and effectiveness of treatment was high, and clinical cure occurred quickly in the patients treated with ITC. These findings agree with the results reported by Queiroz-Telles et al. (2006), who found an efficacy of 100% and an effectiveness of 94% when ITC was used [13]. Nevertheless, previous studies reported different results. Naranjo et al. [11] assessed 47 patients with PCM and found substantial clinical improvement in 89% of the sample, but clinical cure in only 2% during the first six months of treatment. In a randomized clinical trial of 14 patients with PCM, Shikanai-Yasuda et al. found complete cure in 57% of the sample and clinical improvement in 43% after six months of treatment with ITC [16]. Those discrepancies might be explained by the lower daily doses of ITC (50–100 mg) used in the last two studies quoted, in addition to a shorter time of follow-up (six months at most), thus resulting in a large number of individuals who had partial improvement but did not achieve clinical cure. Because the effect of ITC is dose-dependent [25], higher doses might potentiate the antifungal effect and thus allow for faster clinical improvement. In the present study, ITC was prescribed in daily doses of 200 mg, which might account for the high rate of clinical cure attained in a shorter time compared with the previous studies.

The high rates of efficacy and effectiveness exhibited by treatment with CMX are similar to those reported by other studies, which varied from 62 to 100% [6], [20], [26]; however, a discrepancy was found in the time to achieve clinical cure. In the present study, the median time to clinical cure with CMX was 162 days, while Barbosa and Vasconcelos found that recovery occurred in up to 35 days [6]. It should be observed that those authors established lesion regression as the single criterion for the assessment of the clinical response, while the present study included several other parameters as criteria of cure, including recovery of the disposition to work and of body weight, which might account for such substantial discrepancy.

The efficacy and effectiveness of both ITC and CMX in the initial stage of treatment were highly satisfactory. Although three patients exhibited therapeutic failure, and two had to discontinue treatment because of side effects in the group that used CMX, no difference was found between both groups. This finding lends further support to the recommendations made by the Brazilian consensus on the treatment of PCM [17]. Nevertheless, treatment with ITC or CMX differed as to the speed of its effect. Among the individuals with the chronic form of disease, clinical cure was achieved faster by the ones treated with ITC compared to the ones treated with CMX. The relevance of this novel and original finding might be fully appreciated when one takes into consideration that 75 to 90% of the individuals with PCM exhibit its chronic form; the use of ITC will reduce the length of treatment and thus increase treatment adherence.

Some changes occurred in the shift from initial to complementary treatment because some volunteers discontinued treatment after they achieved clinical cure. In addition, ITC was changed to CMX in some cases during the complementary stage because CMX is distributed gratis at public health care centers.

The group treated with ITC followed by CMX was younger compared with the remainder of the groups. This difference notwithstanding, ITC and CMX exhibited high efficacy in the complementary treatment without differences between them; thus, the recommendations made by the Brazilian consensus on PCM are justified [17]. However, because some patients from both groups discontinued treatment and side effects occurred in the patients treated with CMX, the therapeutic success rate decreased, resulting in a level of effectiveness lower than the level of efficacy.

There are no differences of efficacy and effectiveness of complementary treatment between ITC and CMX in this study. Queiroz-Telles et al (1998) also observed no difference between these two antifungal compounds and the same efficacy we observed [18]. Borges et al (2014) also compared these antifungal compounds with four years of follow up and observed that ITC (86,4%) had higher effectiveness than CMX (51,3%) to reach serological cure [19]. However, they did not perform a homogeneity analysis between two groups. These authors affirmed that severe patients were treated intravenous CMX until clinical improvement followed by oral therapy with CMX. This indicates that the CMX group had patients more severe than ITC group which could justify this difference. In our study we determined a sulfamethoxazole serum levels during initial and complementary treatment which could improve the effectiveness of CMX because this allowed to managing drug dose and enhanced adherence to therapy.

There are few studies assessing long-term treatment with ITC or the time to achieve serologic cure. Shikanai-Yasuda et al. found a significant reduction of the serum-specific antibody titers after 10 months of treatment with ITC [16], and Naranjo et al. found a similar reduction in six patients who were followed for more than 12 months [11]. Nevertheless, none of those studies reported on the time elapsed until serologic cure was achieved. Regarding CMX, Valle et al. (1993) found serologic cure in 72% of the cases in their study; however, neither of the above studies commented on the time that elapsed before that outcome was attained [20].

Borges et al observed a lower time to achieve serologic cure to ITC (12 months) than CMX (23 months) [19]. Similar result was found by Queiroz-Telles et al which the ITC group reached serologic cure with 7 months and CMX group with 24 months [18]. In our study the time to achieve serologic cure did not differ between the groups treated with ITC (5 months) or CMX (17 months). A small number of patients that used ITC in complementary treatment could explain the lack of difference between these groups. Furthermore, because individuals with PCM experience depressed cell-mediated immunity [27] and stimulation of antigen-dependent antibody production [28], the serologic cure seems to depend on the recovery of the individual's immune response, rather than on the direct action of the antifungal drugs. The latter seem to act by reducing the fungal load, thus favoring the reestablishment of the immune balance, which is a late occurrence [29]. Perhaps this phenomenon is independent from the particular type of antifungal drug used. The tendency toward faster achievement of a serologic cure exhibited by the patients with the chronic form of disease who were treated with ITC might be related to the fact that their initial antibody titers were lower.

The efficacy and effectiveness of treatment with ITC followed by CMX did not differ compared with treatment with ITC or CMX alone. However, the time to achieve serologic cure is noteworthy, as it was much longer in the group treated with ITC followed by CMX than in the patients treated with ITC or CMX alone, particularly in the patients with the chronic form of the disease. Further studies are needed to explain that finding, including a higher number of patients treated with ITC as initial and CMX as complementary treatment.

The incidence of individuals who attained apparent cure was high, thus demonstrating the high effectiveness of the investigated drugs in the treatment of PCM. The fact that reactivations tend to occur much later has been shown by previous studies, although the present study did not aim to assess reactivation.

Both ITC and CMX proved to be safe in the treatment of PCM, and the incidence of side effects did not differ between them. Only a small number of the patients who used CMX required changes in their treatment as a function of side effects. The individuals treated with ITC reported headache, dizziness, and epigastric pain as the main side effects of treatment. Previous studies assessing ITC described few patient-reported side effects, including headache, exanthema, and epigastric pain and alterations in liver enzymes [11], [13], [16]. The incidence of clinically manifested side effects exhibited by the patients treated with CMX was higher because of the high frequency of epigastric pain.

ESR and the markers of active inflammation decreased as a result of antifungal treatment. The time elapsed until the return of those variables to their normal values was closer to the time to the clinical cure than to the time to serologic cure, thus confirming their status as useful criteria for the assessment of the early response to treatment, as the present study hypothesized.

The important consideration is the poor penetration of ITC through the blood brain barrier. In this study only three patients had central nervous system involvement. In these cases the CMX was the drug of choice.

Considering the period between the introduction and the discontinuation of the treatment, which are different for the antifungal compounds, the cost of the medication is 2.8 higher for ITC than CMX when the reference listed drug is used, and 1.6 higher if the generic is prescribed. However, CMX is distributed free of charge by the Brazilian government while ITC is not.

Although the investigators did not interfere in the choice of antifungal regimen, the present was not a randomized double-blind study, which would have provided a higher level of evidence relative to the assessment of the investigated treatments. Therefore, the study design represents a limitation of the present study.

The present study showed that the individuals treated with ITC achieved clinical cure earlier and exhibited better tolerance of treatment compared with those treated with CMX. Because it is administered in one single daily dose and must be taken after a meal, ITC is more practical than CMX. Nevertheless, a more thorough assessment of the complementary treatment is called for. The findings of the present study allow ITC to be considered as the first-choice antifungal agent for the treatment of PCM.
